# Supplementary material for: A Humanized Yeast Phenomic Model of Deoxycytidine Kinase to Predict Genetic Buffering of Nucleoside Analog Cytotoxicity
Source: Genes (Basel). 2019 Sep 30;10(10):770. doi: 10.3390/genes10100770 (PMC6826991; doi:10.3390/genes10100770)

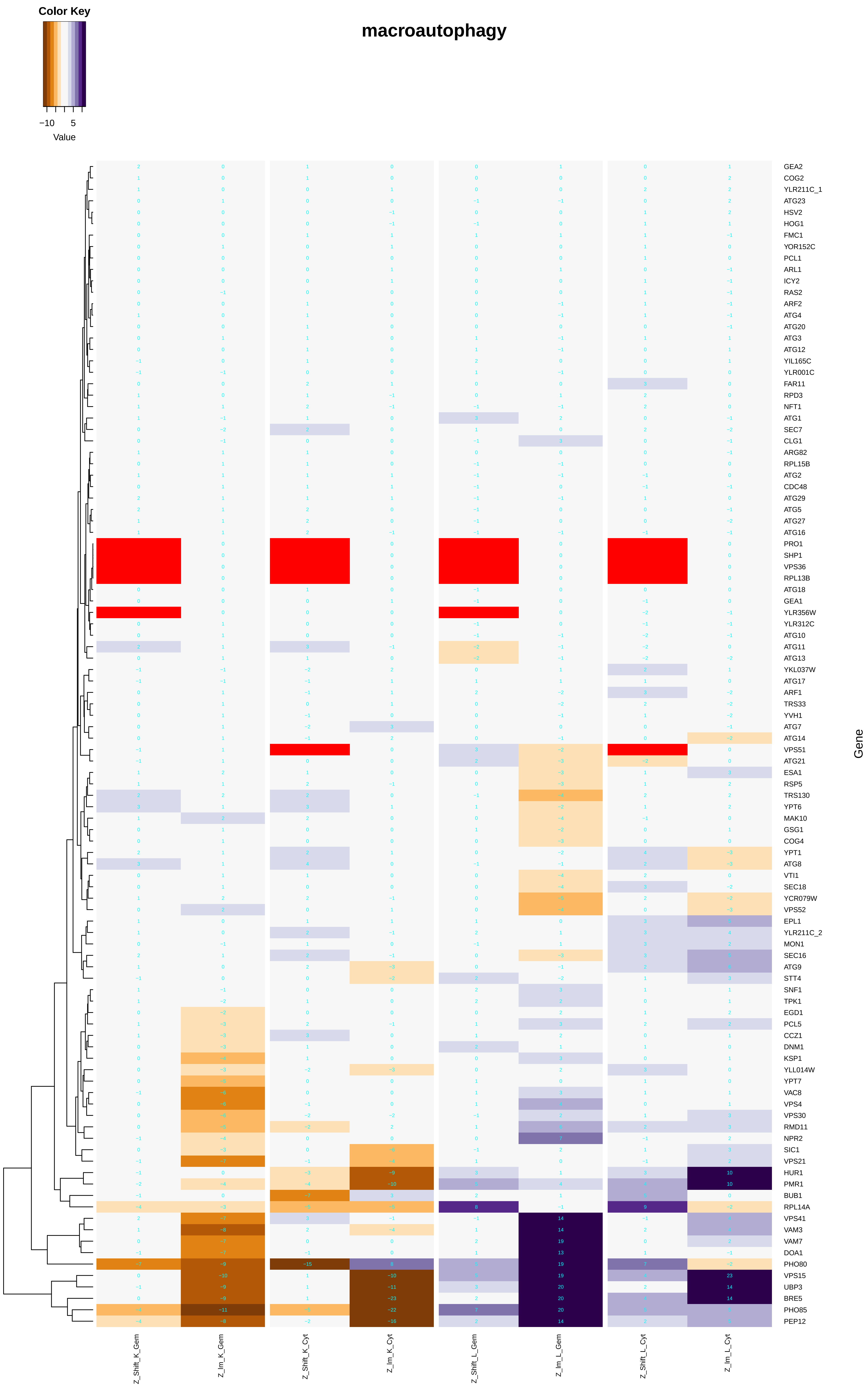

macroautophagy
Color Key
−10
5
Value
GEA2
COG2
YLR211C_1
ATG23
HSV2
HOG1
FMC1
YOR152C
PCL1
ARL1
ICY2
RAS2
ARF2
ATG4
ATG20
ATG3
ATG12
YIL165C
YLR001C
FAR11
RPD3
NFT1
ATG1
SEC7
CLG1
ARG82
RPL15B
ATG2
CDC48
ATG29
ATG5
ATG27
ATG16
PRO1
SHP1
VPS36
RPL13B
ATG18
GEA1
YLR356W
YLR312C
ATG10
ATG11
ATG13
YKL037W
ATG17
ARF1
TRS33
YVH1
ATG7
ATG14
VPS51
ATG21
ESA1
RSP5
TRS130
YPT6
MAK10
GSG1
COG4
YPT1
ATG8
VTI1
SEC18
YCR079W
VPS52
EPL1
YLR211C_2
MON1
SEC16
ATG9
STT4
SNF1
TPK1
EGD1
PCL5
CCZ1
DNM1
KSP1
YLL014W
YPT7
VAC8
VPS4
VPS30
RMD11
NPR2
SIC1
VPS21
HUR1
PMR1
BUB1
RPL14A
VPS41
VAM3
VAM7
DOA1
PHO80
VPS15
UBP3
BRE5
PHO85
PEP12
Gene
Z_Shift_K_Gem
Z_Im_K_Gem
Z_Shift_K_Cyt
Z_Im_K_Cyt
Z_Shift_L_Gem
Z_Im_L_Gem
Z_Shift_L_Cyt
Z_Im_L_Cyt

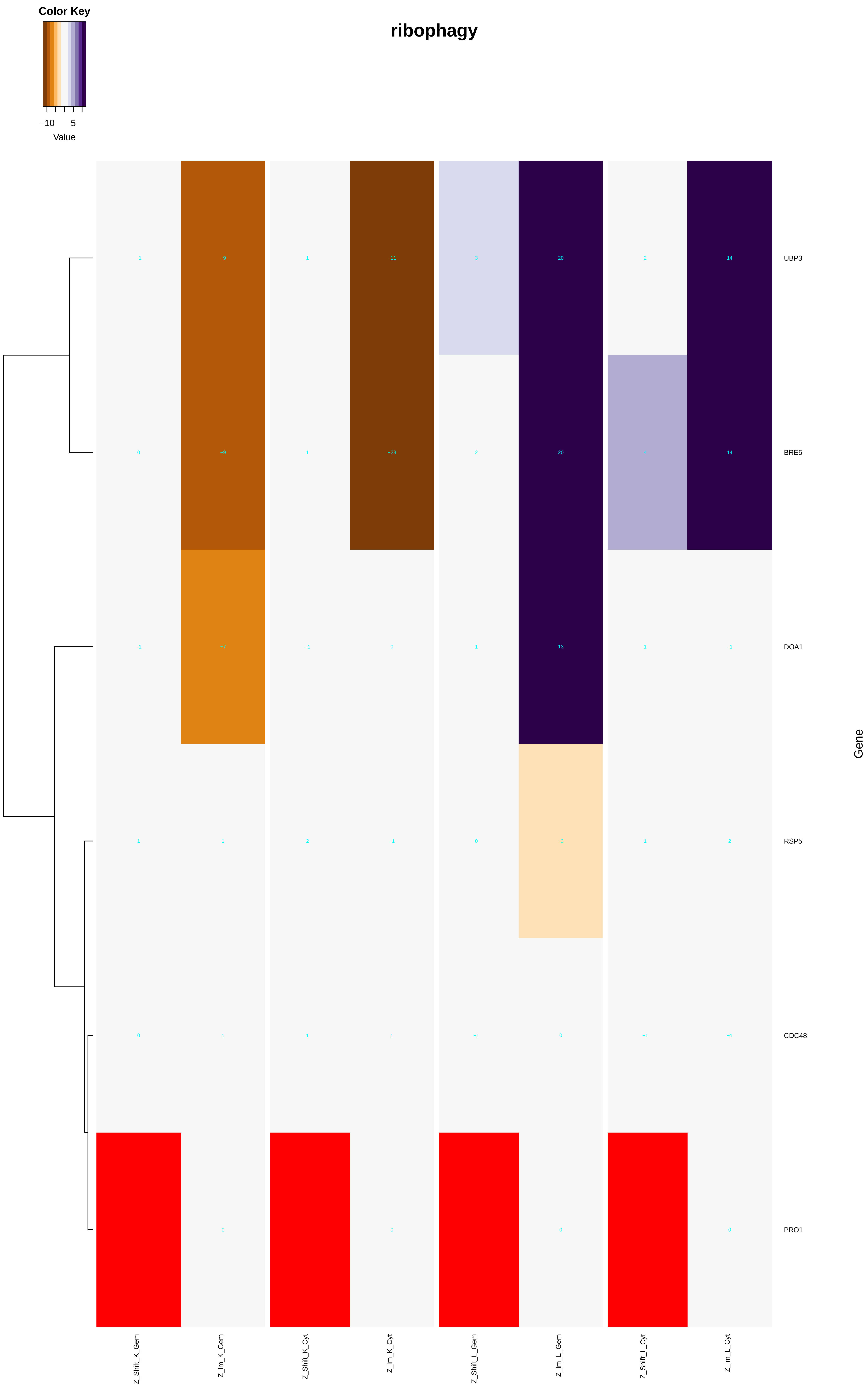
ribophagy
Color Key
−10
5
Value
UBP3
BRE5
DOA1
RSP5
CDC48
PRO1
Gene
Z_Shift_K_Gem
Z_Im_K_Gem
Z_Shift_K_Cyt
Z_Im_K_Cyt
Z_Shift_L_Gem
Z_Im_L_Gem
Z_Shift_L_Cyt
Z_Im_L_Cyt

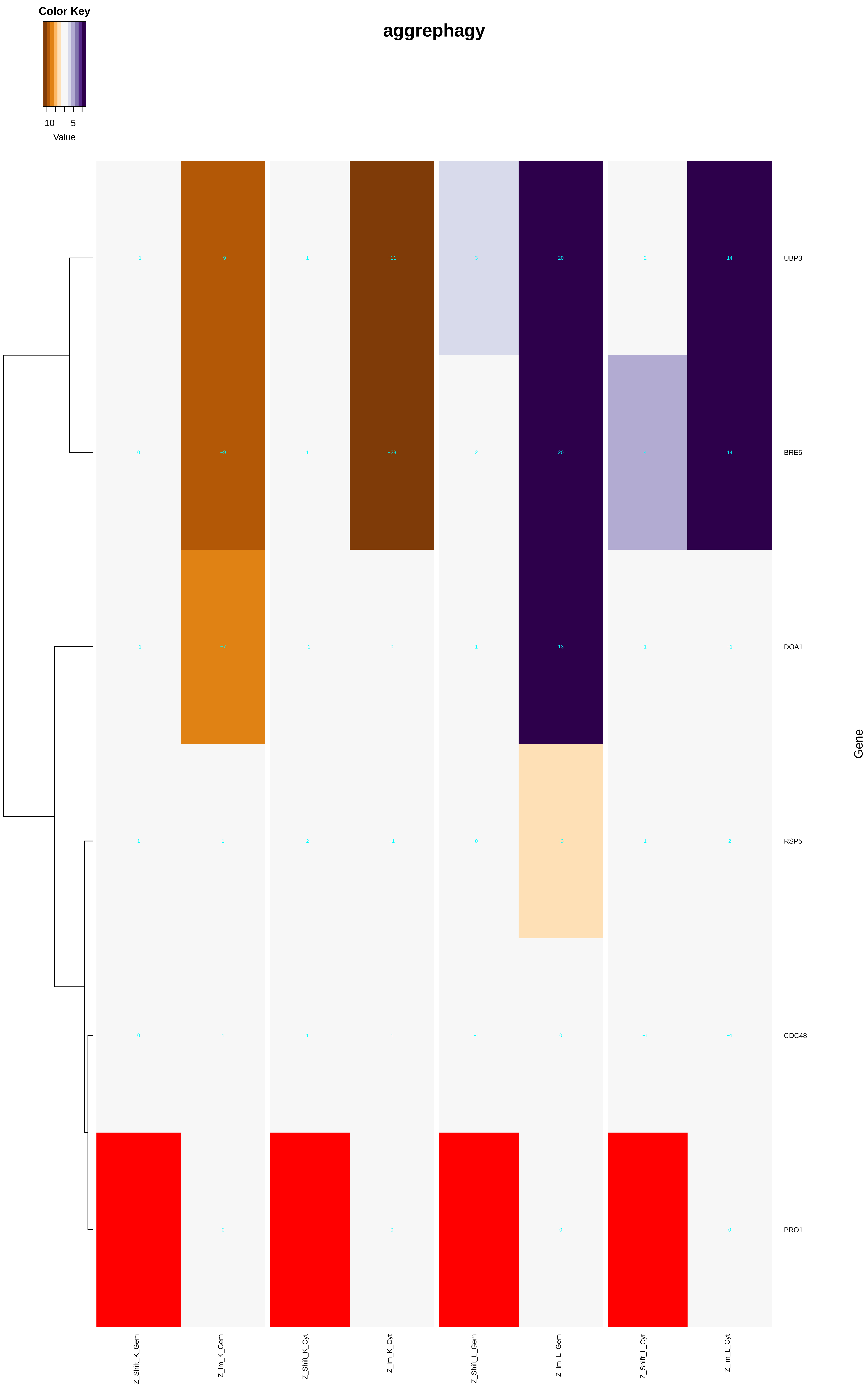
Color Key
−10
5
Value
aggrephagy
UBP3
BRE5
DOA1
RSP5
CDC48
PRO1
Gene
Z_Shift_K_Gem
Z_Im_K_Gem
Z_Shift_K_Cyt
Z_Im_K_Cyt
Z_Shift_L_Gem
Z_Im_L_Gem
Z_Shift_L_Cyt
Z_Im_L_Cyt

reticulophagy

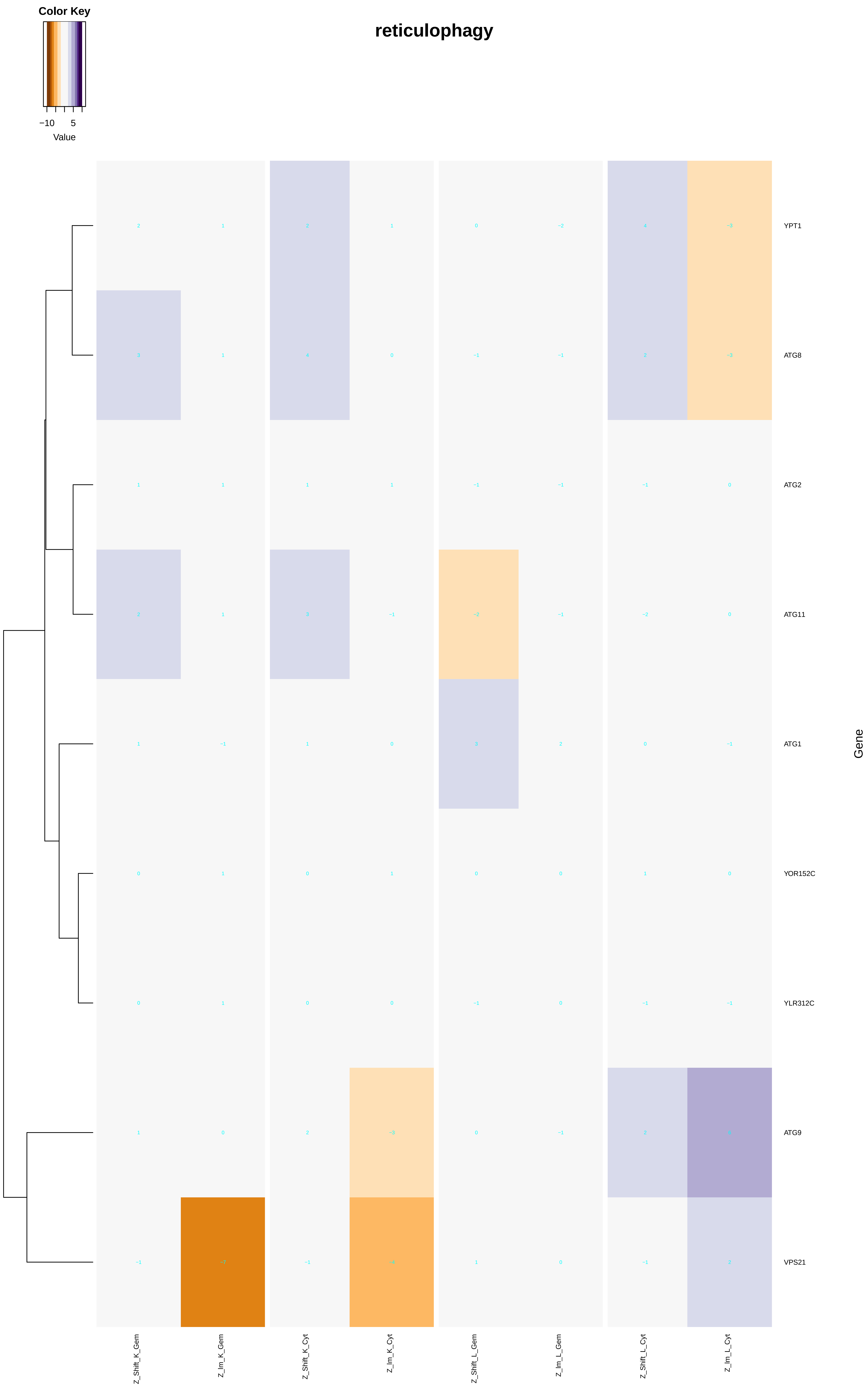

Supplement: Supplementary file 1 [file genes-10-00770-s001.zip › Additional_Files/Additional_File7_GOTermHeatmaps.zip!/Additional_File7_GOTermHeatmaps/macroautophagy.pdf]
